# Prevalence of Dysglycemia Among Coronary Artery Bypass Surgery Patients with No Previous Diabetic History

**DOI:** 10.1186/1749-8090-6-104

**Published:** 2011-09-02

**Authors:** Joseph T McGinn, Masood A Shariff, Tariq M Bhat, Basem Azab, William J Molloy, Elaena Quattrocchi, Mina Farid, Ann M Eichorn, Yosef D Dlugacz, Robert A Silverman

**Affiliations:** 1 Cardiothoracic Surgery Department, Heart Institute at Staten Island University Hospital, 475 Seaview Ave, Staten Island, New York, USA; 2 State University of New York Health Science Center at Brooklyn, 450 Clarkson Avenue, Brooklyn, New York, USA; 3 Department of Internal Medicine, Staten Island University Hospital, 475 Seaview Ave, Staten Island, New York, USA; 4 Pharmacy Clinic, Long Island University, Brooklyn, New York, USA; 5 Krasnoff Quality Management Institute, North Shore-Long Island Jewish Health System, 600 Northern Boulevard, Great Neck, New York, USA; 6 Department of Emergency Medicine, Long Island Jewish Medical Center, North Shore-Long Island Jewish Health System, 270-05 76th Avenue, New Hyde Park, New York, USA; 7 Hofstra North Shore-LIJ School of Medicine, Hofstra University, Hempstead, New York, USA

**Keywords:** HbA1c, coronary artery bypass grafting (CABG), coronary artery disease (CAD), dysglycemia, increased risk of diabetes, diabetes

## Abstract

**Background:**

Dysglycemia is a major risk factor for atherosclerosis. In many patient populations dysglycemia is under-diagnosed. Patients with severe coronary artery disease commonly have dysglycemia and there is growing evidence that dysglycemia, irrespective of underlying history of diabetes, is associated with adverse outcome in coronary artery bypass graft (CABG) surgery patients, including longer hospital stay, wound infections, and higher mortality. As HbA1c is an easy and reliable way of checking for dysglycemia we routinely screen all patients undergoing CABG for elevations in HbA1c. Our hypothesis was that a substantial number of patients with dysglycemia that could be identified at the time of cardiothoracic surgery despite having no apparent history of diabetes.

**Methods:**

1045 consecutive patients undergoing CABG between 2007 and 2009 had HbA1c measured pre-operatively. The 2010 American Diabetes Association (ADA) diagnostic guidelines were used to categorize patients with no known history of diabetes as having diabetes (HbA1c ≥ 6.5%) or increased risk for diabetes (HbA1c 5.7-6.4%).

**Results:**

Of the 1045 patients with pre-operative HbA1c measurements, 40% (n = 415) had a known history of diabetes and 60% (n = 630) had no known history of diabetes. For the 630 patients with no known diabetic history: 207 (32.9%) had a normal HbA1c (< 5.7%); 356 (56.5%) had an HbA1c falling in the increased risk for diabetes range (5.7-6.4%); and 67 (10.6%) had an HbA1c in the diabetes range (6.5% or higher). In this study the only conventional risk factor that was predictive of high HbA1c was BMI. We also found a high HbA1c irrespective of history of DM was associated with severe coronary artery disease as indicated by the number of vessels revascularized.

**Conclusion:**

Among individuals undergoing CABG with no known history of diabetes, there is a substantial amount of undiagnosed dysglycemia. Even though labeling these patients as "diabetic" or "increased risk for diabetes" remains controversial in terms of perioperative management, pre-operative screening could lead to appropriate post-operative follow up to mitigate short-term adverse outcome and provide high priority medical referrals of this at risk population.

## Background

Diabetes Mellitus (DM) is a major risk factor for the development of vascular disease, including coronary artery disease (CAD) [1-6]. Still, up to one third of patients with diabetes remain undiagnosed and may remain so for years, since the slowly progressing dysglycemic phase of this disease does not typically produce symptoms [7]. Earlier identification of asymptomatic dysglycemic patients may lead to more timely interventions and treatment to prevent or delay end-organ damage, and life-style modification and/or medication may reduce the progression of increased risk for diabetes into diabetes [8].

Identifying dysglycemia may be accomplished with an overnight fasting blood glucose, a two hour oral glucose tolerance test, and more recently the American Diabetes Association (ADA) indicated hemoglobin A1C (HbA1c) testing is an acceptable alternative for diagnosing dysglycemia [9]. The HbA1c has the following advantages over glucose based tests: it is not necessary to fast before testing; the test can be obtained in acute care settings; and the time of day of the blood drawn is not relevant.

Patients with coronary artery disease are at a high risk for having dysglycemia. There is growing evidence that dysglycemia irrespective of underlying history of diabetes is associated with adverse outcomes in coronary artery bypass graft (CABG) surgery patients, including increased length of stay, wound infections and higher mortality [10-12]. We propose that pre-operative assessment of patients undergoing CABG surgery presents an opportunity for identifying adults with previously undiagnosed dysglycemia and might lead to appropriate post-operative follow up to mitigate short-term adverse outcome and high priority medical referral of this at risk population since earlier diagnosis of diabetes can potentially prevent long-term complications. The goal of this study was to determine the prevalence of undiagnosed dysglycemia as defined by the HbA1c level among patients undergoing CABG.

## Methods

Patients presenting to a community tertiary hospital for cardiac surgery between the years 2007 and 2009 had HbA1c assayed during the pre-operative assessment. According to our surgical protocol HbA1c is routinely obtained pre-operatively, whether patients have a known history of diabetes or not. Patients were included if they were undergoing CABG with or without valve replacement and had a pre-operative HbA1c value recorded. Data was retrospectively obtained from the medical charts and the center's Centricity Cardiology Data Management System Society of Thoracic Surgeons (STS) Adult Cardiac database (version 2.52.1 and 2.61). The HbA1c was assayed in the Staten Island University Hospital clinical laboratory using the Tosoh G7 analyzer (Tokyo, Japan).

The main objective of this study was to determine the prevalence of dysglycemia in patients undergoing CABG who had no known history of diabetes. We used the 2010 ADA guidelines for HbA1c identification of patients with potential dysglycemia [9]. A HbA1c of 6.5% or higher categorizes patients as having "diabetes", a HbA1c of 5.7-6.4% categorizes patients as "increased risk for diabetes" and a HbA1c level less than 5.7% is considered normal. We also determined the total prevalence of "diabetes" and "increased risk for diabetes" in all patients undergoing CABG by combining patients with known diabetes into the total count. Descriptive statistics, chi-square and analysis of variance were used to analyze the data; analyses were conducted using Statistical Analysis System (SAS) version 9.2. A *p*-value equal to 0.05 was considered statistically significant. The study was approved by the Staten Island University Institutional Review Board.

## Results

1157 patients underwent CABG during the three year study period of which 1045 had a pre-operative HbA1c level documented in the medical records. Of the 1045 patients with a recorded HbA1c, 415/1045 (40%) had a known history of DM and 630/1045 (60%) had no known history of DM (Table 1). The distribution of HbA1c for all 1045 patients is found in Table 2.

**Table 1:** Patient characteristics of overall CABG sample and with no known history of diabetes mellitus

	N = 1045	n = 630*
Age (years)	Mean 64.9 (± 10.9)	Mean 65.4 (± 11.1)

Gender (female, %)	258 (24.7%)	148 (23.5%)

Race	Caucasian 902 (86.3%)	Caucasian 571 (90.6%)

	Black 38 (3.6%)	Black 19 (3%)

	Asian 22 (2.1%)	Asian 9 (1.4%)

	Other 71 (6.8%)	Other 29 (4.6%)

Insurance Status	Medicare 527 (50.4%)	Medicare 326 (51.8%)

	Commercial 399 (38.2%)	Commercial 237 (37.6%)

	Medicaid 100 (9.6%)	Medicaid 55 (8.7%)

	Other 19 (1.8%)	Other 12 (1.9%)

Current Smoker	427 (40.9%)	280 (44.4%)

Hypertension	854 (81.7%)	486 (77.1%)

Peripheral Vascular Disease	119 (11.4%)	58 (9.2%)

Myocardial Infarction	499 (47.8%)	288 (45.7%)

Congestive Heart Failure	144 (13.8%)	75 (11.9%)

Minimally Invasive CABG**	334 (32%)	232 (36.8%)

Median Sternotomy CABG	711 (68%)	398 (63.2%)

Emergent Procedure	26 (2.5%)	15 (2.4%)

Urgent Procedure	749 (71.7%)	450 (71.4%)

Elective Procedure	270 (25.8%)	165 (26.2%)

Number of Vessels Revascularized	Mean 3.2 ± 1.2	Mean 3.1 ± 1.2

BMI kg/m^2^***	Mean 29.3 ± 5.7	Mean 28.3 ± 5.2

	Underweight 8 (0.8%)	Underweight 3 (0.5%)

	Normal 238 (22.9%)	Normal 173 (27.6%)

	Overweight 385 (37%)	Overweight 247 (39.4%)

	Obese 410 (39.4%)	Obese 204 (32.5%)

BMI - Body Mass Index; CABG - Coronary Artery Bypass Graft; *No known previous history of diabetes; **Coronary Artery Bypass Grafting via small left thoracotomy; ***Underweight = less than 16.5 kg/m^2^, Normal = 16.4-18.4 kg/m^2^, Overweight = 18.5-24.9 kg/m^2^, Obese = 25.0 kg/m^2 ^and above.

**Table 2:** Frequency of normal and elevated HbA1c among all CABG patients and among those CABG patients with no known history of diabetes

Pre-operative HbA1c Category	All Patients (N = 1,045)	No known history of DM (n = 630)
< 5.7%	222 (21.2%)	207 (32.9%)

5.7-6.4%	439 (42.0%)	356 (56.5%)

≥ 6.5%	384 (36.8%)	67 (10.6%)

DM - Diabetes mellitus.

The following results are for the 630 patients with no known history of DM. The average patient age was 65.4 years, 148/630 (24%) were female and 571/630 (91%) were white. Table 1 includes additional patient characteristics and medical history. The subjects included in this study had an average body mass index (BMI) of 28.3 kg/m^2^, 77% had a history of hypertension, and 46% had a previous myocardial infarction. The mean number of vessels surgically revascularized in this group was 3.1.

The main study findings are reported in Table 2. A total of 207/630 (32.9%) patients were found to have a HbA1c in the normal range, 356/630 (56.5%) had an HbA1c in the "increased risk for diabetes" range and 67 (10.6%) patients had an HbA1c in the diabetes range.

Table 3 indicates the relationship between selected risk factors and HbA1c. We found no differences in age, gender, insurance status, or personal medical history between the normal and study determined pre-diabetic and diabetic groups. The proportion of patients with a history of myocardial infarction increased with increasing HbA1c (40.6% of those in the normal category and 50.8% of those in the diabetic category), although this trend was not significant. The mean BMI increased significantly with increasing HbA1c, and those whose HbA1c indicated diabetes had more vessels revascularized (mean 3.6) than did those in the normal and pre-diabetic groups (mean 3.1, p = 0.009).

**Table 3:** Comparison of clinical variables by HbA1c in patients without known diabetes (n = 630)

HbA1c (%)	No DM < 5.7 (n = 207)	Increased risk for DM 5.7-6.4 (n = 356)	DM ≥ 6.5 (n = 67)	*P *value
Age (years)	Mean 64.6 ± 12.3	Mean 65.8 ± 10.6	Mean 64.9 ± 9.9	0.592

Female	38 (18.4%)	89 (25.0%)	21 (31.3%)	**0.055**

Male	169 (81.6%)	267 (75.0%)	46 (68.7%)	

Race	Caucasian 195 (94.0%)	Caucasian 316 (88.8%)	Caucasian 60 (89.6%)	0.970

	Black 3 (1.5%)	Black 14 (3.9%)	Black 2 (3.0%)	

	Asian 0	Asian 7 (2.0%)	Asian 2 (3.0%)	

	Other 9 (4.4%)	Other 19 (5.3%)	Other 1 (1.5%)	

Insurance Status	Medicare 101 (48.8%)	Medicare 194 (54.5%)	Medicare 31 (46.3%)	0.479

	Commercial 82 (39.6%)	Commercial 127 (35.7%)	Commercial 28 (41.8%)	

	Medicaid 18 (8.7%)	Medicaid 29 (8.2%)	Medicaid 8 (11.9%)	

	Other 6 (2.9%)	Other 6 (1.7%)	Other 0	

Current Smoker	92 (44.4%)	160 (45.0%)	28 (41.8%)	0.893

Hypertension	158 (76.3)	277 (77.8%)	51 (76.1%)	0.902

Peripheral Vascular Disease	21 (10.1%)	29 (8.2%)	8 (11.9%)	0.523

Myocardial Infarction	84 (40.6%)	170 (47.8%)	34 (50.8%)	0.176

Congestive Heart Failure	24 (11.6%)	38 (10.7%)	13 (19.4%)	0.127

Dyslipidemia	148 (71.5%)	265 (74.4%)	52 (77.6%)	0.564

Minimally Invasive CABG*	83 (40.1%)	131 (36.0%)	18 (26.9%)	0.149

Median Sternotomy CABG	124 (59.9%)	225 (63.2%)	49 (73.1%)	0.149

Emergent Procedure	3 (1.5%)	9 (2.5%)	3 (4.5%)	

Urgent Procedure	153 (73.9%)	243 (68.3%)	54 (80.6%)	

Elective Procedure	51 (24.6%)	104 (29.2%)	10 (15.0%)	0.420

Number of Vessels Revascularized	Mean 3.1 ± 1.14	Mean 3.1 ± 1.14	Mean 3.6 ± 1.16	**0.009**

BMI (%)	Mean 27.7 ± 4.9	Mean 28.5 ± 5.4	Mean 29.5 ± 5.4	**0.031**

BMI - Body Mass Index; CABG - Coronary Artery Bypass Graft; DM - Diabetes Mellitus; *Coronary Artery Bypass Grafting via small left thoracotomy.

We identified the scope of dysglycemia among all 1045 patients undergoing CABG, which included patients with a known history of diabetes. A total of 482/1045 (46%) of all patients undergoing CABG were identifiable as having diabetes, including the 415 known diabetic patients and the 67 newly discovered diabetic patients. On evaluation of the frequency of dysglycemia in patients undergoing CABG, we found that 838/1045 (80%) of all patients had either a history of known diabetes or an elevated HbA1c (>5.7%) at the time of surgery.

## Discussion

We found undiagnosed dysglycemia is common among patients having coronary revascularization surgery, with 67% of patients newly diagnosed at the time of CABG surgery. This includes 57% of patients meeting the criteria for increased risk for diabetes (HbA1c 5.7-6.4%) and 11% of patients meeting criteria for diabetes (HbA1c greater or equal to 6.5%). Recent evidence that dysglycemia irrespective of underlying history of diabetes is associated with adverse outcome in CABG patients, including longer hospital stay, wound infections and higher mortality, may by itself justify the need for screening [10-12]. This is in addition to any long term benefit provided by earlier medical referral and management of newly diagnosed patients. The study data also highlights the very common association of dysglycemia with coronary artery disease, since when including the previously and newly diagnosed patients, a total of 80% of all patients undergoing CABG have dysglycemia.

Our findings that elevated HbA1c levels are common among patients requiring surgical revascularization with no known history of diabetes are supported by large studies of patients presenting with acute coronary syndrome, where a consistently high frequency of undiagnosed dysglycemia using glucose-based testing has been reported [1,13-15]. Based on the strong association of symptomatic CAD and diabetes, the European Society of Cardiology recommends diabetes screening for all patients hospitalized with acute coronary syndrome [16]. There are fewer studies that report screening for undiagnosed diabetes or pre-diabetes among patients undergoing CABG. In Sweden, among 267 patients undergoing CABG and without a known diabetes history, 73% were found by oral glucose tolerance testing (OGTT) to have either pre-diabetes or diabetes [17]. In a report from Turkey of 166 patients undergoing CABG, 60% of those without a diabetes history were diagnosed with dysglycemia using the OGTT [18]. In a study measuring HbA1c levels among 163 non-diabetic patients undergoing CABG, HbA1c was 6.0% or higher in 93/163 (57%) patients and 7% or higher in 19/163 (12%) patients [19]. Our study, using a larger sample size, is consistent with these findings and indicates substantial case-finding when the HbA1c is used to identify abnormalities. Still, to our knowledge, there has not been a national or global systematic effort to promote either HbA1c or glucose based testing among patients undergoing coronary bypass surgery.

In a recent report of National Health and Nutrition Examination Survey (NHANES) data, the frequency of Hba1c newly identified DM among the USA adult population was 1.8%, a rate much lower than our findings [20]. This difference may in part be explained by the high frequency of multiple risk factors for pre-DM and DM in our study population. Still, it is not clear why newly diagnosed diabetic patients undergoing CABG have not been previously informed of having diabetes, especially since chronic medical problems such as hypertension, hyperlipidemia and coronary artery disease were fairly common. Most the study patients had health care insurance, and would have the potential to access and use primary and preventive care. It is possible that the inconvenience and preparation needed for glucose based testing leads to missed cases and fewer diagnoses. The recent ADA recommendation of using HbA1c as an alternative test to identify diabetes, which does not require an overnight fast and can be obtained at any time, may provide greater opportunity for diagnosis in a wider range of clinical settings and eventually lead to fewer undiagnosed individuals.

Diabetes screening most typically occurs during outpatient primary or medical care visits, particularly since long term follow-up and care will be needed. As our findings suggest, this should not preclude screening in a high risk population in an acute care setting. Patients presenting for cardiac surgery could be screened pre-operatively and the results used to inform the treating surgeon of undiagnosed dysglycemia. As the evidence of HbA1c as a short-term prognostic marker in CABG patients grows pre-operative HbA1c testing may be needed to optimize perioperative care and prevention of post-operative complications; this remains to be prospectively investigated. In addition, further study is needed to determine if pre-operative normalization of the HbA1c confers short or long-term post-operative benefit.

Post-operatively, patients with abnormal HbA1c could be referred for outpatient follow-up, a medical consult or a session with a diabetes nurse educator could take place prior to discharge. Emphasis on the management of other contributory comorbiditites in this population may further help in preventing progression of underlying medical disease. While counseling the management of chronic disease may be challenging in acute care settings, individuals will sometimes show greater interest in their health during times of illness and opportunities for early diagnosis should not be lost. During a brief discussion patients with elevated HbA1c could be encouraged to partner with a provider and maintain long term care as well as attempt life-style modifications. The concept of the 'teachable moment' has been demonstrated in the case of smoking cessation - patients are more likely to quit smoking following health events, such as pregnancy, hospitalizations, or diagnosis of cancer [21]. Such health events represent opportunities for health care providers to educate patients and encourage behavior modifications. Medical triggers are associated with better short- and long-term weight loss, which could be one component of a diabetes intervention [22].

## Conclusion

In conclusion, our study found a high prevalence of dysglycemia in patients undergoing CABG without a prior history of diabetes. These findings suggest that all patients with no known history of diabetes undergoing CABG should be screened for dysglycemia, particularly as it has been recently reported as an independent risk for post-operative complications and adverse outcomes. Further, an abnormal HbA1c warrants an inpatient or post-discharge medical referral to initiate treatment and help prevent long-term complications.

## List of abbreviations

CAD: Coronary Artery Disease; CABG: Coronary Artery Bypass Grafting; ADA: American Diabetes Association; DM: Diabetes mellitus; HbA1c: Hemoglobin A1C; STS: Society of Thoracic Surgeons; BMI: Body Mass Index; OGTT: Oral Glucose Tolerance Testing

## Competing interests

The authors declare that they have no competing interests.

## Authors' contributions

RAS developed the study concept, supervised the data analysis and drafted the manuscript. JTM designed the protocol, contributed to the discussion, and reviewed/edited the manuscript. MAS researched the data, contributed to the discussion, and reviewed/edited the manuscript. AME analyzed and researched data. MF collected data and reviewed/edited the manuscript. TMB, BA, EQ, WJM, and YDD contributed to the discussion and reviewed/edited the manuscript.

All authors read and approved the final manuscript.
